# What Makes the Gut-Lung Axis Working? From the Perspective of Microbiota and Traditional Chinese Medicine

**DOI:** 10.1155/2024/8640014

**Published:** 2024-01-18

**Authors:** Hui Wang, Ying Wang

**Affiliations:** Zhejiang Chinese Medical University, Hangzhou 310000, China

## Abstract

**Background:**

An increasing number of studies have proved that gut microbiota is involved in the occurrence and development of various lung diseases and can interact with the diseased lung. The concept of the gut-lung axis (GLA) provides a new idea for the subsequent clinical treatment of lung diseases through human microbiota. This review aims to summarize the microbiota in the lung and gut and the interaction between them from the perspectives of traditional Chinese medicine and modern medicine.

**Method:**

We conducted a literature search by using the search terms “GLA,” “gut microbiota,” “spleen,” and “Chinese medicine” in the databases PubMed, Web of Science, and CNKI. We then explored the mechanism of action of the gut-lung axis from traditional Chinese medicine and modern medicine.

**Results:**

The lung and gut microbiota enable the GLA to function through immune regulation, while metabolites of the gut microbiota also play an important role. The spleen can improve the gut microbiota to achieve the regulation of the GLA.

**Conclusion:**

Improving the gut microbiota through qi supplementation and spleen fortification provides a new approach to the clinical treatment of lung diseases by regulating the GLA. Currently, our understanding of the GLA is limited, and more research is needed to explain its working principle.

## 1. Introduction

Human-related microbes are made up of fungi, bacteria, viruses, protozoa, and archaea [1]. The gut is the organ with the most abundant microbes within the human body. Microbiota in the gut is viewed as a sophisticated ecosystem that plays a crucial part in maintaining human health [2, 3]. As sequencing technologies have developed, we have started to acquire an overall perspective on what makes up the microbial community [4]. According to statistics, approximately 1,000 species of bacteria colonize the gut, vastly outnumbering human genes [5]. Recent studies have shown that the gut microbiota is closely related to the occurrence and development of diseases in various systems of the whole body, and terms such as the “gut-brain axis,” “gut-liver axis,” and “GLA” have been proposed. From the perspective of TCM, the spleen is the “mother” of the lung. The function of the spleen can affect the composition of the gut microbiota. The gut microbiota can be improved through qi supplementation and spleen fortification to achieve the regulation on the GLA. The current research of the GLA in various literature studies is not comprehensive. In this review, we summarize the current state of the GLA from the perspective of lung and gut microbiota and their interactions, as well as from the perspective of traditional Chinese medicine (TCM), to provide new ideas for clinical research.

## 2. The Microbiota and Its Effect in the Gut and Lung Microenvironment

### 2.1. The Gut Microbiota and Its Regulatory Effect

The gut microbiota starts to build up after birth and stabilizes after three years of age [6]. The gut microbiota is dominated by Bacteroidetes, Proteobacteria, Actinobacteria, and Firmicutes [7, 8]. Meanwhile, changes in the gut microbiota are also influenced by the mode of delivery. Compared to vaginal delivery, infants born by cesarean section have higher abundance of *Bifidobacterium* and Bacteroides [9]. When variations in the composition of the microbiota are divided into clusters or groups, they are called enterotypes, which can be used as a highly effective way to distinguish human gut microbiota. People with different enterotypes may metabolize and store energy differently [10]. For example, Bacteroides tend to break down carbohydrates; therefore, people with this enterotype are less likely to gain weight. At present, there is great interest in the study of Bacteroides in gut microbiology. Studies have proved that human diseases are related to differences in the gut microbiota. Gut microbiota and its metabolites not only regulate human health but also play a vital role as a bridge between diet and host [11, 12]. Epidemiology studies suggest that food and eating habits shape gut microbiota [13]. The unstable gut microbiota during its formation is prone to metabolic and immune diseases, and a single-type diet that reduces gut microbiota leads to aging and weakness in the elderly [14, 15]. Antibiotics can also disrupt the gut microbiota, causing an imbalance in gut homeostasis [16].

With the development of fecal microbiota transplantation (FMT), there are new methods in modern medicine for treating various diseases caused by gut microbiota disorders and the importance of normal microbiota in humans has received increasing attention [17, 18]. Currently, FMT is the most effective method for treating Clostridioides difficile infection [19, 20]. A recent case report found favorable outcomes for FMT in patients with recurrent Clostridium difficile infection and coexisting COVID-19 [21]. According to studies, prostaglandin G2, leukotriene B4, and corticosterone are contained in the supernatant of fecal microorganisms. These metabolites have proinflammatory effects and can be removed by washing, a method that effectively improves the safety of treatment [22]. In addition to FMT, probiotics also improve the gut microbiota. Probiotics are living microbes, mainly Lactobacillus and Bifidobacterium, that maintain the health of the host when consumed in sufficient quantities [23]. Studies have confirmed that probiotics can promote gut health, which are mainly used to inhibit pathogenic bacteria, and maintain mucosal immune homeostasis and epithelial function [24]. Researchers induced rats and found that there was a decrease in the ratio of Firmicutes to Bacteroidetes content in aging rats, which increased significantly after *Lactobacillus fermentum* and *Lactobacillus helveticus* were administered [25]. The researchers administered nutrients with the above properties to obese patients, and the results showed that the host's metabolism increased and the gut microbiota changed [26].

### 2.2. The Lung Microbiota and Its Regulatory Effect

Compared with that of the gut microbiota, at present, the study of the lung microbiota is in its infancy. The respiratory tract is made up of two parts and is used for gas exchange. Historically, the lung has been considered sterile for a long time, mainly because of the challenges in cultivating the lung microbiota of healthy people via routine microbial cultures [27]. This theory was not questioned until microbial DNA was detected in the lungs of healthy individuals through sequencing [28, 29]. Lung microbiota belongs to the low biomass microbiota, and its makeup is susceptible to the external environment [30]. The balance among the relative growth rate, migration, and elimination of lung microorganisms is the prerequisite for the formation of the lung microbiota [31]. Studies have shown that the upper respiratory tract has the highest concentration of bacteria, inhibiting the spread of respiratory pathogens [32]. Upper airway microbiota is derived from microaspiration [33]. The presence of lung microbiota, which is present in the lungs at 11 weeks of age, shows DNA signatures. The lung microbiota mainly consists of Firmicutes, Proteobacteria, Bacteroidetes, and Actinobacteria [34].

The respiratory tract microbiota of the patients differs from that of healthy individuals [28]. Numerous subsequent studies have confirmed this observation [35]. Recent evidence has revealed that the lower respiratory tract microbiota also affects lung health [36, 37]. The lower respiratory tract shares the same predominant microbial composition as the gastrointestinal tract, including Firmicutes and Bacteroidetes [38, 39]. Some scholars believed that the lung microbiota is the key to lung cancer (LC) immunotherapy [40]. Dietary fermentable fiber content changes the body's microbiota, which is characterized by Bacteroidetes and Firmicutes, to reshape the immune environment of the lung [41]. Likewise, smoking alters the bacterial community in the lung, especially Streptococcus, Campylobacter, and Prevotella content [42]. Smoking comes into direct contact with the airways and passes through the lungs, stimulating an inflammatory response in the lungs and impairing their defenses, which can lead to changes in the types of microbiota in the lungs [43].

## 3. The Microbiota and GLA

### 3.1. GLA and the Interaction between the Gut and the Lung

The respiratory tract and gut have separate physiological functions and environments, but their similar structures and shared embryonic origins provide the basis for the link between the two. Both the lung and trachea develop from the foregut of the archenteron, and the respiratory and glandular epithelium is differentiated from the endoderm of the gut. The microbiota maintains the dynamic balance of the human body and promotes normal physiological functions, and the microbiota in different parts are interconnected. In addition to causing gastrointestinal diseases, an imbalance in gut microbiota will also cause pulmonary diseases and vice versa. The crosstalk between the lung and the gut microbiota is called the GLA [44]. This particular research field has attracted much attention.

### 3.2. Gut-Lung Crosstalk Leads to Simultaneous Lung and Gut Diseases

Recent experimental studies have demonstrated the gut-lung crosstalk [45, 46]. In clinical observations, patients with lung disease showed poor appetite, constipation, and loose stools, indicating the link and characteristics of simultaneous lung and large intestine diseases [47, 48]. When untreated mice were compared with mice with sepsis, the diversity of microbiota in the lung of the mice with sepsis increased remarkably, including numerous bacteria from the gut, such as Bacteroides [31]. One of the typical examples here is the coronavirus disease-19 (COVID-19), causing gastrointestinal symptoms. In contrast, COVID-19 may also be prevented through the regulation of gut microbiota [49, 50]. Patients with COVID-19 being treated with probiotics presented a significant relief of gastrointestinal symptoms such as nausea and vomiting; meanwhile, the probiotic treatment also reduced the risk of respiratory damage [49]. Another study discovered that chronic obstructive pulmonary disease (COPD) increases the incidence of inflammatory bowel disease (IBD), and IBD increases mortality in patients with COPD [51]. The researchers assessed 273208 COPD patients with Cox proportional hazards models, and the result (hazard ratio 1.23, 95% CI 1.09–1.4) verified that IBD increased the risk of death in patients with COPD [51]. In addition, other studies have also made similar conclusions. For example, compared with healthy people, patients with COPD are 2-3 times more likely to have IBD, and half of the patients with IBD will develop lung disease [52, 53]. A study found that microbiota in the upper airway and gut improve the body's resistance to lung infections and concluded that lung and gut microbiota are essential factors in maintaining lung health [54]. When mice lungs were stimulated with lipopolysaccharides, there was a remarkable increase in the number of gut bacteria [55]. The synchronization of the changes in the lung and gut is an embodiment of their mutual communication.

### 3.3. Microbiota May Play a Crucial Role in Establishing GLA

Numerous studies have shown that microbiota plays an essential part in the interaction between the lung and the gut [8]. Research has revealed that the imbalance of Firmicutes and Bacteroidetes in the gut of rats can aggravate acute lung injury caused by sepsis. When the ratio returns to normal, acute lung injury is relieved [56]. Changes in gut microbiota are significantly correlated with lung microbiota as confirmed in a study of patients with HIV pneumonia, and evidence of microbial translocation has been found [57]. Preliminary results from a study of cystic fibrosis and the GLA suggest that the content of gut microbiota and upper airway microbiota is characterized by a simultaneous increase in the number of their constituents [58]. In another study, gut microbiota was shown to modulate respiratory infections and susceptibility to viruses [59]. The subjects were divided into healthy and COPD groups, showing different microbiota [60]. The diversity of gut microbiota is reduced in children with recurrent respiratory infections and tuberculosis [61]. Due to the differences in the pathogenic bacteria related to respiratory tract infections, the gut microbiota also undergoes certain changes [62]. Thus, the microbiota is crucial for the functioning of the GLA.

#### 3.3.1. The Lung and Gut Local Immunomodulatory Regulation Is Interconnected through Microbiota

The mechanism of gut-lung crosstalk is complex. The correlation between microbiota and immune function has been extensively studied and confirmed [62]. The lung and gut microbiota are important for immune regulation, as well as influencing disease development and prognosis. Changes in the composition and function of gut microbiota affect the respiratory system through the common mucosal immune system [8, 63]. Similarly, disturbances in the respiratory microbiota can also influence the digestive tract by affecting immune regulation [55]. Lung and gut local immunomodulatory regulation is interconnected, in which the common function is to induce IgA production, cytotoxic response, and Th cells [8, 63]. IgA antibodies not only coordinate the balance between bacteria and the host but also neutralize pathogens present in the lung and gut [64]. Lung infection is accompanied by gut damage with altered gut microbiota, which is mediated by Th17 cells [65, 66]. One study found that segmented filamentous bacteria increase Th17 cell proportions in the gut; therefore, it is possible that the gut microbiota also affects the lung through Th17 cells [67, 68].

The lung and the large intestine share common physiological and pathological mechanisms, one of which is mucosal immunity and the migration and homing of innate lymphocytes. Immune cells located in the intestinal mucosa can migrate to distant respiratory mucosa after activation and play an immunomodulatory role [43]. Immune dysregulation and altered microbiota are closely linked to the downregulation of anti-inflammatory molecules like interleukin-4 (IL-4) and upregulation of inflammatory molecules like IL-6 [69, 70]. Studies have shown that gut microbiota composition correlates with plasma concentrations of chemokines, inflammatory cytokines, and markers of tissue damage [71]. Previous research indicated that the gut can recruit CD4+ T cells derived from the lung under the influence of the CCL25/CCR9 axis, disrupting the gut immune system [72]. Gut microbiota can induce the production of the granulocyte-macrophage colony-stimulating factor (GM-CSF) through IL-17, enhancing the defensive capacity of the lung, while Nod2 receptors from gut microbiota can regulate lung immune homeostasis [73].

Gut microbiota dysbiosis causes severe damage to dendritic progenitor cells, monocytes, and lung immunity. The dendritic cells are the antigen-presenting cells that effectively activate T cells, so it is believed that the dendritic cells bridge adaptive and innate immunity in lung mucosal immunity [74, 75]. Dendritic cells in the lung express a large number of pattern recognition receptors and interact with local microbiota to maintain the immune homeostasis of the lung [76]. In addition, symbiotic microbiota therapy can induce the production of intestinal innate lymphoid cells (ILCs), which can increase the level of neutrophils and ultimately improve the immunoregulation of the respiratory system [62]. One study demonstrated that ILCs can be recruited from the gut to the lungs in response to inflammatory signals [77]. In diseases such as asthma and COPD, the identification of the ILCs in the GLA reflects the linking of gut microbiota to the lung [78].

Gut microbiota enhances the phagocytosis of primary alveolar macrophages and plays a protective role during pneumonia [79]. Rifaximin is an antibiotic that can effectively ameliorate lung and gut damage caused by the influenza virus. It reduces lung and gut permeability by enhancing tight junction protein expression, thus maintaining microbiota stability [80]. Additionally, food reflux or microinhalation can direct gut microbiota to the lungs to mediate immune responses [81, 82].

#### 3.3.2. Gut Microbiota Metabolites, as Important Immunomodulators, Participate in the Lung Immune Response

Microbiota studies in the gut have explored the gut microbiota metabolites and how they relate to the immune response in the lung [83, 84]. Short-chain fatty acids (SCFAs) are well-studied immunomodulatory metabolites [85, 86]. They reprogram the metabolism and bind with the lung immune cell receptors to regulate the immune response and enhance the antiviral response of the lung. SCFAs are composed of propionate, pentanoate, acetate, and butyrate and are produced via dietary fiber fermentation [87, 88]. SCFAs act as signal transducers, also known as second messengers, that affect disease progression [89]. SCFAs regulate the gut barrier through histone deacetylase inhibition and G-protein-coupled receptor (GPCR) activation, which are the well-established signaling pathways for SCFA function [90]. Unfortunately, it has not yet been identified which GPCRs have priority in different environments. The most studied GPCRs are GPR109A, GPR41, and GPR43 [91]. In a previous study on mice, it was found that low concentrations of SCFAs increase allergic diseases in the lung, while high concentrations inhibit allergic inflammation in the airways [41].

In addition to SCFAs, long-chain fatty acids (LCFAs) also play an important role. LCFAs are mainly derived from microbial oil, canola oil, soybean oil, and corn oil [92]. An interesting phenomenon is that both *ω*-3 polyunsaturated fatty acids (PUFAs) and *ω*-6 PUFAs are LCFAs; the former promotes anti-inflammatory response, while the latter promotes proinflammatory response [93]. A recent study on non-small-cell lung cancer (NSCLC) showed that under glucose starvation, the platelet isoform of phosphofructokinase 1 (PFKP) promotes LCFA oxidation by phosphorylating the metabolic enzyme acetyl-CoA carboxylase 2 (ACC2) and ultimately promotes the survival of cancer cells [94]. Another study found that LCFA levels are significantly associated with progression-free survival and overall survival in patients with NSCLC [93].

**Figure 1:**
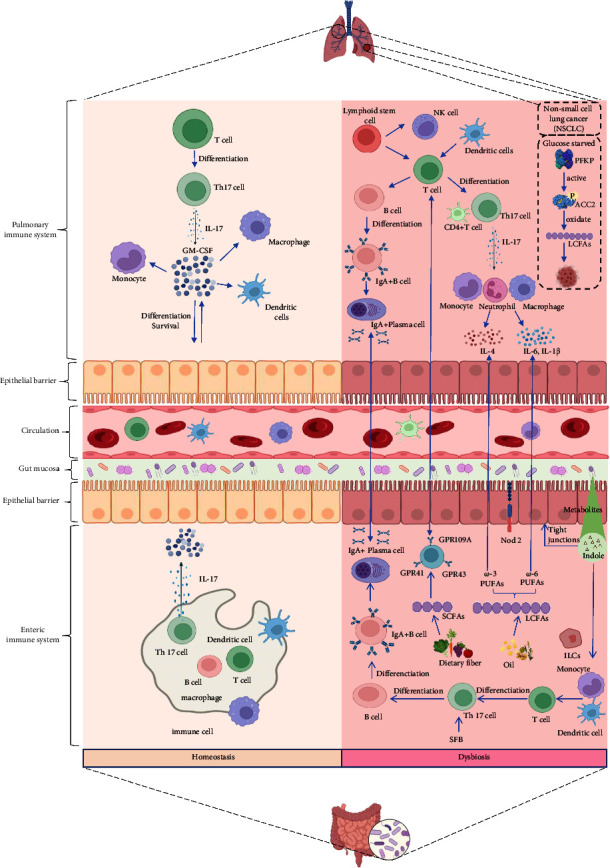
The microbiota plays a crucial role in shaping GLA in terms of immunity.

Furthermore, indole is also a metabolite of gut microorganisms, which restores the gut barrier and activates immune cells [95]. The protective effect of indole depends on aryl hydrocarbon receptor signaling [95]. Based on common mucosal immunity, histamine secreted by gut microbiota can affect lung immunity [96]. This effect of histamine is associated with host histamine receptors and histamine-degrading enzymes [96]. Other metabolites of gut microbiota, such as lipopolysaccharides and bile acids, can also enter the lung through lymphatic or blood vessels to initiate immune response (Figure 1) [70, 97]. Toll-like receptor (TLR)-4 recognizes lipopolysaccharide, a significant component of Gram-negative bacteria's outer membrane and a critical player in the onset of the pulmonary inflammatory response [70]. It has been suggested that bile acids have anti-inflammatory qualities and can block proinflammatory cytokine transcription that is NF-*κ*B-dependent through the farnesoid X receptor (FXR) and membrane G-protein-coupled bile acid receptor GPBAR-1 [98].

Lung and gut local immunomodulatory regulation is interconnected. Lung infection is accompanied by gut damage with altered GM, which is mediated by Th17 cells. SFBs increase Th17 cells in the gut, so it is possible that the GM also affects the lung through Th17 cells. Immune cells located in the intestinal mucosa can migrate to distant respiratory mucosa after activation and play an immunomodulatory role. Immune dysregulation and altered microbiota are closely linked to the downregulation of anti-inflammatory pathways like IL-4 and upregulation of inflammatory pathways like IL-6. GM can induce the production of the granulocyte-macrophage colony-stimulating factor (GM-CSF) through interleukin-17, enhancing the defense capacity of the lung, while Nod2 receptors from GM can regulate lung immune homeostasis. SCFAs regulate the gut barrier through the inhibition of histone deacetylase and the activation of G-protein-coupled receptors (GPCRs). Both *ω*-3 polyunsaturated fatty acids (PUFAs) and *ω*-6 PUFAs are LCFAs; the former promotes anti-inflammatory response, while the latter promotes proinflammatory response.

## 4. The GLA, from the Perspective of TCM

In TCM, rather than referring to the anatomical structures, internal organs are described through visceral manifestation, a special term in TCM, to understand the internal organs through holistic observation and detect the viscera with manifestation. In the viscera-state doctrine, the physiological function of one zang organ or fu organ may involve the physiological function of several organs in the perspective of anatomy, while the physiological function of one anatomic organ can be demonstrated by some zang and fu organs in the viscera-state doctrine.

### 4.1. The Spleen, Lung, and Gut Are Tightly Connected

#### 4.1.1. The Lung and the Large Intestine Have an Interior and Exterior Relationship

In TCM, each zang organ is paired with a fu organ; hereby, the lung is paired with the large intestine [99]. In physiology, a free flow of the lung qi can maintain a normal descent of the bowel movement, while a healthy descent of qi in the large intestine ensures balanced respiration [100, 101]. In pathology, the lung and the large intestine are influenced mutually and diseases involving both the lung and the large intestine are quite common in TCM clinics [102]. Failure of the lung descent may affect the qi of the large intestine, resulting in abnormal bowel movement with symptoms such as constipation and abdominal distension. Similarly, disturbed movement of intestinal qi affects the ascent and descent of the lung, leading to symptoms such as shortness of breath, coughing, expectoration, and blocked feeling of respiration [101].

#### 4.1.2. The Spleen and the Lung Have a Mother-Child Relationship

The spleen in TCM is quite different from that in modern medicine. From the perspective of the Five Elements Theory in TCM, the spleen and the lung are attributed to the earth and metal, respectively [103]. The spleen, as the mother, can intergenerate its child, the lung [104]. The spleen can be regarded as the mother to “offer” nutrients, while the lung as the child to “utilize” the nutrients [105]. In pathology, if spleen qi is deficient, as the mother spleen does not generate the child lung, it will often lead to a deficiency of lung qi. Meanwhile, if lung disease lasts for a long time, as the child lung continuously consumes qi from its mother spleen, it will also lead to spleen qi deficiency, manifesting as shortness of breath, abdominal distension, and other symptoms [103]. Besides, disharmony between the lung and the spleen leads to pathological products such as phlegm, rheum, and edema.

#### 4.1.3. The Spleen, Stomach, and Gut Have an Interior and Exterior Relationship

Similar to the relationship between the lung and the large intestine, the spleen and the stomach also build up an exterior-interior relationship. The spleen is a zang organ which is for storage and is located relatively interior, and the stomach is a fu organ, which breaks things down and is located relatively exterior. They are connected through the spleen and the stomach channels. In qi movement, the spleen qi ascends, while the stomach qi descends, and their ascent-descent coordination is considered as the pivot to the whole body's qi movement. Concerning the relationship between the spleen and the gut, TCM believes that the stomach and the gut are in the same family, and they share many similarities in physiological function. The theory that both the large intestine and the small intestine belong to the stomach was first recorded in Spiritual Pivot-Chapter 2: Root transport, indicating “The large intestine and small intestine linking or pertaining with the foot yangming stomach channel” [106]. Therefore, the spleen-stomach relationship might be extended to the relationship among the spleen, stomach, and gut. Modern research has confirmed that the three are composed of a serosal layer, muscular layer, submucosa, and mucosal layer, and all have digestion, absorption, and secretion functions [106].

### 4.2. The Spleen as Described in TCM May Work Similar to Gut Microbiota in Maintaining the Intestinal Mucosal Barrier

Symptoms such as epigastric and abdominal discomfort, abdominal distension, and diarrhea, which are believed to be caused by the injured intestinal mucosal barrier in modern medicine, actually result from spleen deficiency in TCM [107]. As reported in previous studies on the relationship between intestinal mucosal barrier injury and the spleen-invigorating method, spleen deficiency and intestinal mucosal injury have the same pathophysiological basis; therefore, it is concluded that the normal function of the spleen is the basis of the intestinal mucosal barrier, which might be through gut microbiota [108].

**Figure 2:**
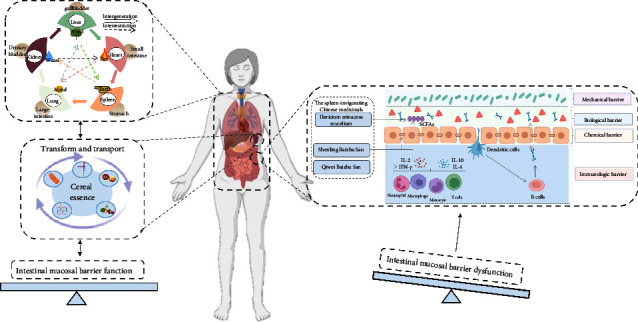
The spleen as described in TCM plays a crucial role in “connecting” GLA.

Many studies have proved that Chinese medicines applied to invigorate the spleen can repair the intestinal mucosal barrier. First, they can promote the expression of tight junction proteins. For example, Shenling Baizhu San can promote the expression of tight junction proteins, such as Claudin, Occludin, JAM, and ZO-1 mRNA, and maintain the integrity of the intestinal mucosal mechanical barrier [109]. Second, they can regulate gut microbiota and its metabolites. Shenling Baizhu San also inhibits the proliferation of pathogenic bacteria, regulates gut microbiota disturbance, and promotes SCFA-producing bacteria such as Adlercreutzia and Clostridium [110]. There is a special polysaccharide in the Hericium erinaceus mycelium that can increase the SCFA content by inhibiting the expression of GPR41 and GPR43 in the colon tissue, which consequently results in a change [111]. Third, they can regulate immunity. Qiwei Baizhu San, as a classic prescription for the treatment of diarrhea, can promote the expression of IL-10, IL-2, IFN-*γ*, and IL-4 in the intestinal mucosal epithelium [112]. Fourth, they can improve intestinal nutrition. It was reported that Sijunzi Tang was used for patients with gastric cancer after surgery to supplement nutrition, and its effect was proved by significant improvement of nutritional indicators, such as serum transferrin and prealbumin, which was regarded as an indication of intestinal mucosal barrier repair (Figure 2) [113].

The spleen governs transportation and transformation. The spleen and lung build up a mother-child relationship, and the spleen is the mother of the lung. The bank up earth to generate metal is to improve the GM by regulating the function of the spleen and the stomach and then affect the colonization of lung microbiota under the action of the GLA, so as to achieve the homeostasis of the lung and the intestine. The normal function of the spleen is the basis of the intestinal mucosal barrier, which might be through gut microbiota. The intestinal mucosal barrier includes the mechanical barrier, biological barrier, and chemical barrier. The spleen-invigorating Chinese medicinals can repair the intestinal mucosal barrier, and its mechanism is mainly manifested in four aspects: first, promote the expression of tight junction proteins; second, regulate the GM and its metabolites; third, regulate immune function; and fourth, supplement intestinal nutrition.

Notes: to make the “connection” easier to be understood, in the middle of Figure 2, we have used a common and simple anatomy diagram to demonstrate the spleen, lung, gut, and other internal organs, but, as mentioned in the article, the zang-fu organs in traditional Chinese medicine are not exactly equal to the anatomical organs in modern medicine.

### 4.3. The Spleen as Described in TCM May Play a Crucial Role in Establishing GLA

Further research proved that spleen dysfunction affects the distribution, type, and content of gut microbiota, resulting in abnormal microbiota and increased harmful bacteria content [114]. For someone who has lung disease, abnormal microbiota and increased harmful bacteria will cause colonization of lung bacteria through the GLA, which consequently leads to the destruction of the body's barrier and decreased immunity, aggravating the lung disease [115]. The above conclusions coincide with the concept of holism in TCM. In humans, the viscera and bowels are an integral part of the overall body function, and the functional activity of any viscera and bowels is an integral part of the overall body function. TCM not only pays attention to the physiological functions of viscera and bowels but also gives great importance to the functional connection and coordination between viscera and bowels, emphasizing that such coordination and connection are related to health and disease.

The spleen and the stomach build up an exterior-interior relationship; meanwhile, the stomach is directly connected to the gut. The lung and the large intestine build up an exterior-interior relationship as well. Furthermore, the spleen and the lung build up a mother-child relationship and the spleen is the mother of the lung. Therefore, the spleen, stomach, lung, and large intestine are bound up in functions. The spleen is the earth that bears and produces everything, including good parts, such as essence and microbiota, as well as bad parts, such as dampness and bacteria. Corresponding to modern medicine, the balance and imbalance of gut microbiota correspond to spleen function in TCM directly. The essence of water and grain transported and transformed by the spleen can be understood as nutrients such as proteins, lipids, carbohydrates, vitamins, and various trace elements [116]. Gut microbiota is an important place for the body to obtain nutrients, participating in the digestion, absorption, and synthesis of nutrients, such as decomposition, protein, lipid, synthesis of various vitamins, and promoting metal ion absorption like iron [116]. Again, the correlation between spleen and gut microbiota has been demonstrated. Therefore, the commonly used method, cultivating the earth (the spleen) to generate the metal (the lung), is actually to improve the gut microbiota by regulating the function of the spleen and the stomach and then affect the colonization of lung microbiota under the action of the GLA, in order to achieve the homeostasis of the lung and the intestine.

## 5. Gut Microbiota Might Be the Hope for the Treatment of Lung Diseases in the Future

The importance of gut microbiota in maintaining human health is self-evident, and numerous studies have confirmed that common lung diseases can be improved by manipulating gut microbiota [117]. Currently, probiotics, drugs, and diet can modulate the gut microbiota, and these have been used in patients with asthma, LC, COVID-19, and tuberculosis [66].

### 5.1. Asthma

Asthma, as a common pulmonary disease, has complicated pathogenic mechanisms [118]. The microbiome analysis has enabled researchers to gain a deeper understanding of asthma [119]. Despite many challenges in the study of lung microbiota, clinical data have revealed a relationship between lung microbiota and asthma [120, 121]. In contrast, changes in gut microbiota can disrupt lung function [122, 123]. Researchers observed that Haemophilus, Moraxella, and Streptococcus are more abundant in the upper airway microbiota of 244 infants from birth to age 5. These genera are associated with an increased risk of chronic wheezing at age 5 [124]. In patients with asthma, the content of Bacteroides, Actinobacteria, and Firmicutes is reduced, while that of Proteobacteria is increased compared to healthy subjects [47]. In experimental neonatal asthma, gut microbiota plays an important role [125]. According to the clinical results, the relative abundance of Faecalibacterium, Lachnospira, Bifidobacteriaceae, Rothia, and Veillonella decreased, along with the decrease of acetate content, indicating the imbalance of gut microbiota [126, 127]. Asthma can be prevented and treated by regulating the respiratory and gut microbiota at an early stage [128, 129]. This was demonstrated by improvements in the gut microbiota in infants at a high risk of asthma who were given Lactobacillus supplementation early in life [130]. Supplements such as probiotics and prebiotics can help reduce asthma symptoms by maintaining a stable gut microbiota [121]. At the same time, azithromycin has the same effect [131]. Pregnant women can improve fetal lung immunity and reduce childhood asthma prevalence through a healthy diet [132]. A healthy diet involves increasing the intake of whole grains, fruits, and vegetables, which have antioxidant properties that can help reduce airway inflammation in asthmatic patients [133]. At the same time, pregnant women should avoid taking a large number of saturated fats, processed foods, and sweetened beverages, which can alter the gut microbiota of the human body, thus reducing the production of SCFAs, and their immune regulation and anti-inflammatory effects may not function properly [132, 133].

In TCM, Liujunzi Tang is used for supplementing qi and fortifying the spleen, which has an impact on gastrointestinal function [134]. In the clinic, Liujunzi Tang has been combined with conventional therapy for the treatment of asthma and had a positive effect. Ginseng and Atractylodis Rhizoma, as the main ingredients in this prescription, are both effective in regulating gut microbiota. Ginseng is involved in qi supplementation and spleen fortification and is used to treat spleen deficiency in rats. Ginseng extract can increase the abundance of Lactobacillus and other probiotics in the rat gut [135]. Lactobacillus can induce the production of interleukins with anti-inflammatory effects that help maintain intestinal barrier function [107]. Atractylodis Rhizoma is commonly used for treating spleen deficiency syndrome. In a study of rats with spleen deficiency, it was found to reduce serum levels of IL-6 and TNF-*α*. 134 IL-6 can be secreted under the induction of TNF-*α* to cause inflammation [136]. At the same time, Atractylodes Rhizoma can regulate the gut microbiota, thereby maintaining normal intestinal permeability through the metabolite butyrate, preventing mucosal inflammation [137].

### 5.2. LC

There are two types of LC, SCLC and NSCLC, which is one of the most fatal malignancies with the fastest-growing mortality and morbidity rate worldwide, among which NSCLC is the most common type [117]. Although immunotherapy and chemotherapy are effective in the treatment of LC, the therapeutic effect is still not satisfactory. With the in-depth study of microbiota, it was found that gut microbiota has an important effect on LC [138]. Researchers examined the gut microbiota of patients with LC using next-generation 16S rRNA sequencing and found differences in microbial composition between patients and healthy people [139]. The abundance of Bifidobacterium and Actinobacterium in patients with LC is lower than that of controls, and the levels of Enterococcus are higher [139, 140]. Another study concluded that compared with healthy controls, Fusobacteria and Bacteroidetes are more abundant in patients with LC, while the abundance of Proteobacteria and Firmicutes is low [141]. Several enriched microbial communities in the lower respiratory tract are thought to be associated with LC, the most abundant of which is the genus Veillonella [142]. Meanwhile, Prevotella, Rothia, and Streptococcus are also abundant in the lower respiratory tract affected by LC [142]. Yogurt and dietary fiber reduce the lung cancer risk as revealed in a large prospective study, which also demonstrates the potential of probiotics and prebiotics to prevent LC [143]. In a study on NSCLC, advanced patients treated with *Clostridium butyricum* had significantly longer overall survival than the controls [144]. Omega-3 fatty acids, a nutrient that reduces inflammation, may also be an option for treating cachexia in LC [145]. A study revealed that berries contain a polyphenol called castalagin, which improves antitumor activity, as well as anti-PD-1 activity, by altering the gut microbiota [146].

Similarly, in TCM, different prescriptions based on the principle of reinforcing the spleen to support the lung combined with other therapies have improved the clinical efficacy in patients with advanced NSCLC, and gastrointestinal symptoms, such as nausea and vomiting, are also relieved [147]. One research revealed that reinforcing the spleen to generate the lung can inhibit the metastasis of NSCLC with different malignancies by downregulating the expression of TNKS2 [148]. Downregulating the expression of TNKS is also one of the possible pathways of gut microbiota in antitumor response [149].

### 5.3. COVID-19

COVID-19 is an infectious disease caused by severe acute respiratory syndrome coronavirus type 2 (SARS-CoV-2) [150]. Apart from the respiratory system, infections also affect the gastrointestinal tract. COVID-19 severity correlates positively with gastrointestinal symptoms. A study of hamsters with COVID-19 found a clear correlation between its severity and changes in the gut microbiota, which is characterized by an increase in the abundance of harmful bacteria and a decrease in SCFA levels and their producers, ultimately leading to changes in the GLA [151]. Researchers examined the gut microbiota of seven patients with COVID-19 without antibiotic treatment and found that the abundance of Clostridium ramosum, the genus Coprobacillus, and *C. hathewayi* from Firmicutes correlates positively with COVID-19 severity. Faecalibacterium, prausnitzii, and Alistipes onderdonkii abundance correlates negatively with COVID-19 severity [152, 153]. Among 20 people who died from COVID-19, it was discovered that enrichment of Acinetobacter abundance in the lung is the main feature of pulmonary microbial homeostasis disorder [154]. In another study, Rothia, Veillonella, and Actinomyces abundance was significantly high in the lungs of patients with COVID-19 [155]. In addition, the lungs of patients often contained Enterobacteriaceae that were prevalent in the gut, which further confirmed the importance of gut-lung crosstalk disorder in COVID-19 [154]. A decreased abundance of Lactobacillus and Bifidobacteria was found in patients with COVID-19. Dietary fiber and probiotics enhance anti-inflammatory and immune responses in patients with COVID-19, especially those with mild or acute symptoms [156]. 150 COVID-19 patients were randomly divided into two groups. The results showed that the remission rate of the patients supplemented with probiotics was significantly higher than that of the control group [50]. The intake of cucumbers and cabbages was negatively correlated with the mortality of patients with COVID-19 [157]. Adding fermented foods such as kombucha, kefir, or foods containing prebiotics and probiotics, as well as diet supplements, can serve as a comprehensive nutritional strategy to better cope with COVID-19, which is achieved by increasing the gut microbiota function and mucosal immunity [158].

COVID-19 belongs to the category of TCM pestilent disease. Professor Zhang Zhiming in China created the Xuan Fei Hua Zhuo Fang, which focuses on strengthening the spleen and the stomach, while regulating the three zang-fu organs of the lung, spleen, and large intestine [159]. This formula has been used in the early stage of COVID-19 with a remarkable curative effect. The No. 3 COVID-19 formula is mainly composed of Sheng Mai San and Si Jun Zi Tang, which can reinforce the spleen to support the lung and treat the deficiency of both the lung and the spleen in the late stage of COVID-19 [160].

### 5.4. Tuberculosis (TB)

Tuberculosis is an infectious respiratory disease caused by Mycobacterium tuberculosis, which has a high incidence rate and high mortality [94]. A previous study found that the gut microbiota imbalance causes the reduction of the number of bacterial species and the decline of immune regulation including the downregulation of IFN-*γ*, a cytokine used to kill Mycobacterium tuberculosis [161]. This result confirms the significant role of GLA in TB. The species with a significantly reduced content in the gut microbiota of patients with active TB is *Akkermansia muciniphila* [94]. In another study, the authors concluded that Bacteroides abundance decreases significantly [161]. Lung microbiota is characterized by a high abundance of Staphylococcus aureus as revealed by a study on patients with TB [162]. Another study showed a high abundance of Lachnospiraceae in the gut of patients with TB [163]. Compared with the control group, Prevotella histicola abundance in the sputum microbiota of patients with TB increased significantly [164]. Probiotics, such as Lactobacillus plantarum, can be used to regulate human immunity and thus inhibit Mycobacterium tuberculosis [76, 165]. This offers a new treatment for TB while demonstrating the potential of probiotics.

TB is a wasting disease that affects immune function. Traditional antituberculosis drugs often have adverse reactions of varying degrees. Exploring new treatment methods is particularly important for the treatment of TB. The clinical efficacy of Fuzheng Kanglao combined with quadruple therapy is significantly good, with IL-10 expression increased, and IL-6 and TNF-*α* expression decreased [166]. White hyacinth bean, lotus seed, poria, and astragalus root have good qi-supplementing and spleen-fortifying effects. In addition, astragalus root can not only regulate the expression of inflammatory factors but also reduce adverse reactions to drugs [166].

Another interesting phenomenon is that the specific phylum of the lung and the gut regularly change in diseases mentioned above (Table 1). For example, in patients with asthma, the abundance of Actinobacteria decreases in both the lung and the gut, while Firmicutes abundance increases in the lung and decreases in the gut. In patients with LC, the abundance of Bacteroidetes increases both in the lung and the gut, while that of Actinobacteria increases in the lung but decreases in the gut. In patients with COVID-19, the abundance of Firmicutes increases both in the lung and the gut, while that of Proteobacteria increases in the lung but decreases in the gut. In patients with TB, the abundance of Firmicutes increases both in the lung and the gut, while as Bacteroidetes grow in the lung, their abundance is declined in the gut. Therefore, in certain lung diseases, variations in the abundances of some phyla are characterized by either varying in the same direction or opposite directions. The conclusion may be accidental, or for certain specific situations, further studies might be necessary to elucidate this phenomenon.

## 6. Discussion

Lung and gut microbiota play an important regulatory role in the establishment of the GLA through immunity, and metabolites of gut microbiota are also indispensable. The gut microbiota is involved in the occurrence and development of various lung diseases and is linked to the pathological state of the lung. From the perspective of TCM, the function of the spleen can affect the composition of the gut microbiota. The gut microbiota directly reflects the state of the spleen. At the same time, the spleen is the “mother” of the lung. Therefore, the gut microbiota can be improved through qi supplementation and spleen fortification to achieve the regulation on the GLA. The concept of GLA provides a new idea for the subsequent clinical treatment of lung diseases, such as asthma, LC, COVID-19, and TB. However, we only have a limited understanding of how it works. Therefore, many studies have not revealed the entire causality, which is an urgent problem to be solved.

At present, most studies on the GLA focus on the influence of gut microbiota on lung diseases, while the research on the influence of the lung on the large intestine has not made substantial progress. TCM accepts the correlation between the large intestine and the lung. Different from the modern research on the GLA, TCM pays more attention to the influence of the lung on the physiological functions of the large intestine, based on the concept of holism, centering on the five zang organs. The integration of TCM and Western medicine is one of the important concepts of integrated medicine, and both Chinese and Western medicines work synergistically rather than as substitutes. This article promotes in-depth research and discussion of GLA from a holistic and multidimensional perspective to improve the curative effect on patients.

## Figures and Tables

**Table 1:** Lung and gut microbiota and common diseases.

Disease	Sample	Phylum
Asthma	Human	Similarities: Actinobacteria ↓↓ Differences: Firmicutes ↑↓ [47, 124, 126, 127]

Lung cancer	Human	Similarities: Bacteroidetes ↑↑ Difference: Actinobacteria ↑↓ [139–142]

COVID-19	Human	Similarities: Firmicutes ↑↑ Differences: Proteobacteria ↑↓ [152–155]

Tuberculosis	Human	Similarities: Firmicutes ↑↑ Differences: Bacteroidetes ↑↓ [161–164]

## Data Availability

All available data have been included in the article.
